# Supplementary material for: High capacity in G protein-coupled receptor signaling
Source: Nat Commun. 2018 Feb 28;9:876. doi: 10.1038/s41467-018-02868-y (PMC5830429; doi:10.1038/s41467-018-02868-y)
Supplement: Supplementary file 1 — Supplementary Info File #1 [file 41467_2018_2868_MOESM1_ESM.pdf]

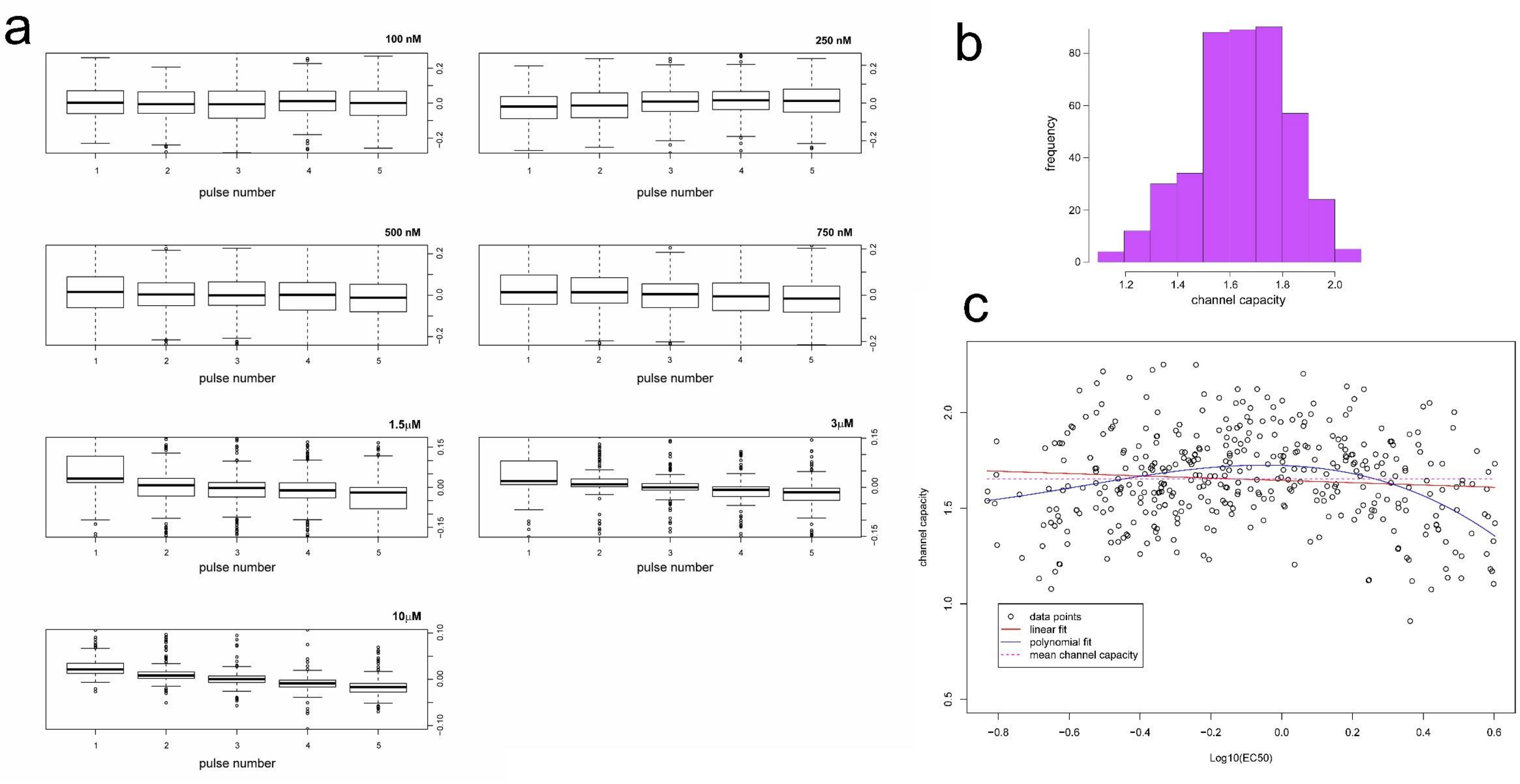
a
100 nM
250 nM
500 nM
750 nM
1.5μM
3μM
10μM
pulse number
b
frequency
channel capacity
c
channel capacity
Log10(EC50)
data points
linear fit
polynomial fit
mean channel capacity

Supplementary Figure 1: Lower bound and EC50 distribution of the channel capacity in the muscarinic acetylcholine receptor-induced calcium response.

(a) Our dose response experiments shows that adaptation occurs at higher Ach concentration, indicating that the channel is not memoryless. Its ability to convey information thus likely exceeds its formal capacity. Boxplots indicate distribution of the residuals as a function of the pulse number. At higher Ach stimulations, the response tends to decrease with each pulse. P-values are significant (< 10^{-4}) for [Ach] = 750nM and above (Kruskal-Wallis test). Correcting for adaptation by centering the boxplot on zero increases the average channel capacity by 6%, indicating that its effect on our capacity estimate are very small. Note that at this experimental setup (7 concentrations given in 5 repeated pulses), no decrease in the peak height is seen at repeated activations by 250nM Ach, unlike with the setup when this concentration was applied 20 times (Fig. 2d). (**b**) Histogram of estimated lower bounds for the channel capacity derived from 433 individual cell's Ca^{2+} response traces. All lower bounds are above 1 and the average estimate is given by 1.65. (**c**) The EC50 was estimated for the response of each cell using sigmoidal model fit (see Supplementary Data 2). The plot shows that channel capacity values are slightly increased for cells showing response with EC50 in the mid-range as indicated by the 3-rd order polynomial fit.

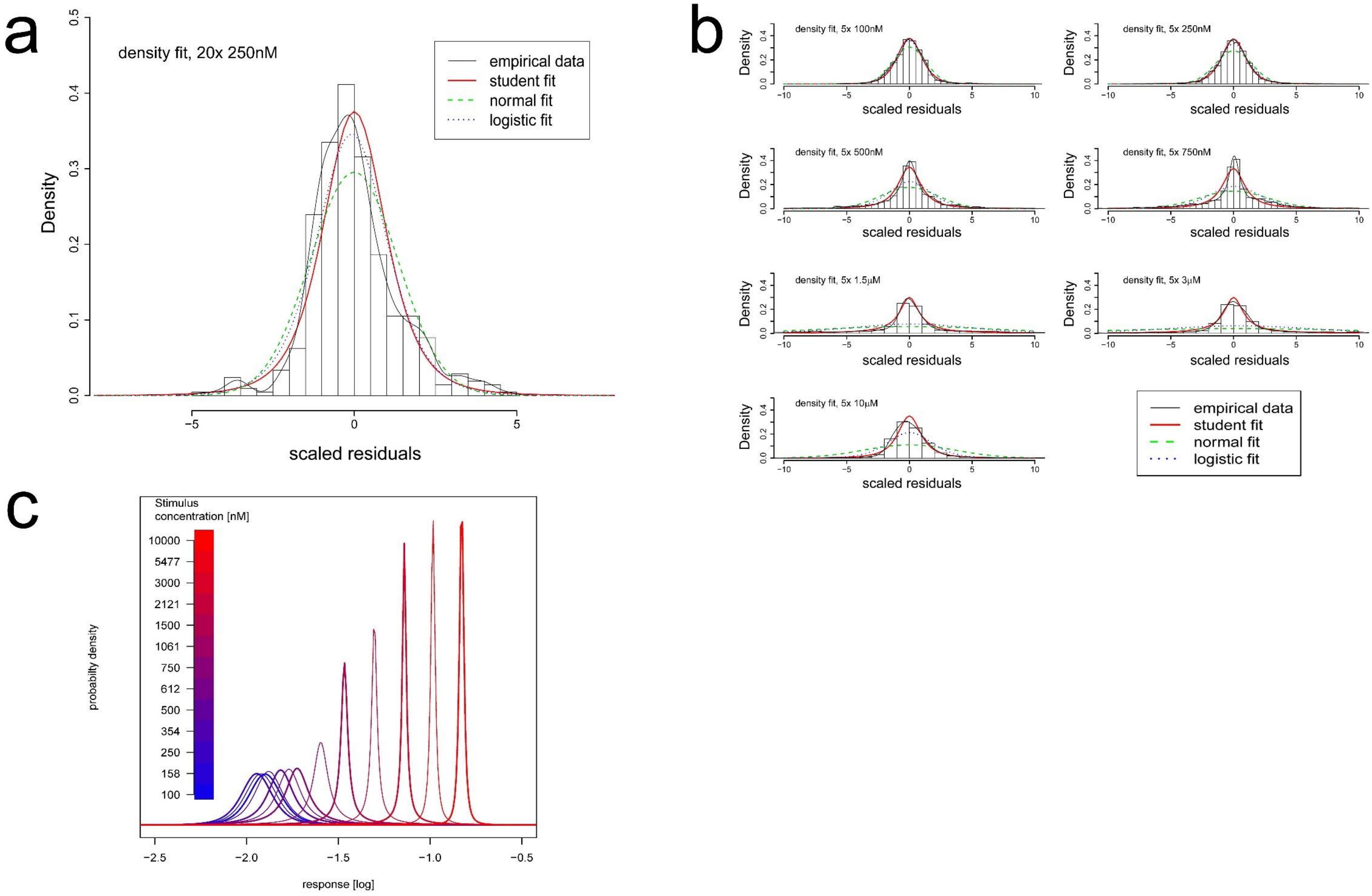
a
density fit, 20x 250nM
empirical data
student fit
normal fit
logistic fit
Density
scaled residuals
b
density fit, 5x 100nM
density fit, 5x 250nM
density fit, 5x 500nM
density fit, 5x 750nM
density fit, 5x 1.5μM
density fit, 5x 3μM
density fit, 5x 10μM
Density
scaled residuals
empirical data
student fit
normal fit
logistic fit
c
Stimulus
concentration [nM]
10000
5477
3000
2121
1500
1061
750
612
500
354
250
158
100
probability density
response [log]

Supplementary Figure 2: Distribution in the muscarinic acetylcholine receptor-induced calcium response.

(a,b) The distribution of the residuals $e_{ik}(c_j)$ is well approximated by a *t*-distribution (Student distribution) but not by a Gaussian distribution. Histograms and thin black lines show the observed distribution of the residuals, and solid red, dashed green and dotted blue lines indicate the fitted distributions for *t*-distributions, Gaussian distributions and logistic distributions, respectively. The fitted *t*-distribution is consistently closer from the empirical distribution than the Gaussian and logistic fitted distributions, both for the adaptation control (20x stimulations at 250nM Ach, **a**) and the escalating dose response experiments (7x concentrations, 5x pulses, **b**). **(c)** Example of interpolation for concentrations not experimentally tested. Thick lines represent the estimated distribution of the response for the tested stimulus concentrations for a representative cell. Thin lines represent the response distribution for untested concentrations obtained through parameter interpolation. The range between two consecutive tested concentrations was discretized with 10 log-spaced values, of which the middle one is shown on the plot (thin lines).
