# Supplementary material for: High capacity in G protein-coupled receptor signaling
Source: Nat Commun. 2018 Feb 28;9:876. doi: 10.1038/s41467-018-02868-y (PMC5830429; doi:10.1038/s41467-018-02868-y)
Supplement: Supplementary file 3 — Description of Additional Supplementary Information [file 41467_2018_2868_MOESM3_ESM.docx]

**Description of Additional Supplementary Files**

File Name: Supplementary Movie 1

Description:

Example of the 70min-long single experiment with seven Ach concentrations used at five repetitions. The video demonstrates course of a single experiment in which cells were repeatedly (n=5) stimulated by 7 different concentrations of the ligand and highlights heterogeneity of the cell responses within a population. The ratio of images recorded for Ca2+-bound and unbound Fura-2 AM (excitation at 340 and 380nm, correspondingly) over the entire experiment was used to create a video. The trace plot of one of the cell and a slider are shown below to visualize a course of the experiment. The color coding of the different ratio values is shown to the right. Image ratio and color conversion was done using ImageJ, the trace and slider were prepared using ImageMagick, the video was compiled and encoded in OpenShot editor at 30 FPS. Background is not removed on purpose, to illustrate how it gradually rises during the experiment.

File Name: Supplementary Data 1

Description:

Ca2+ responses of all the cells (grouped as 27 experiments) used in the current work. The response presented in text files is expressed as a ratio of the Fura-2 AM bound/unbound signals for different cells within a field. Configuration files are generated by manual processing of the traces using script in Supplementary Data 2 and contain information about reference traces and excluded artifacts. They can be used by the script to replicate the peak calling and the height extraction.

File Name: Supplementary Data 2

Description:

MATLAB script which treats files in Supplementary Data 1 to extract the peak heights and estimate channel capacity using alternative estimate of 𝑃𝑖(𝑟|𝑐) by assuming independent Gaussian distributions (see Methods section). The script is currently configured for automatic use of configuration files from Supplementary Data 1.

File Name: Supplementary Data 3

Description:

Peak values obtained for valid traces in each experiment in the Supplementary Data 1 and for the 20 repeated stimulations with 250nM Ach.

File Name: Supplementary Data 4

Description:

R script used to analyze the Supplementary Data 3 and reproducing panels in Figures 2c, 2d, 3d, and Supplementary Figures 1 and 2.
